# Abortion on Request, Contraceptive Access Barriers, and Mental Health-Related Quality of Life Among Women Attending a Romanian Tertiary Center

**DOI:** 10.3390/healthcare14030310

**Published:** 2026-01-26

**Authors:** Bogdan Dumitriu, Flavius George Socol, Ioana Denisa Socol, Lavinia Stelea, Alina Dumitriu, Adrian Gluhovschi

**Affiliations:** 1 Doctoral School, “Victor Babes” University of Medicine and Pharmacy Timisoara, 300041 Timisoara, Romania; bogdan.dumitriu@umft.ro (B.D.); george.socol@umft.ro (F.G.S.); ioana.socol@umft.ro (I.D.S.); 2 Department of Obstetrics and Gynecology, “Victor Babes” University of Medicine and Pharmacy Timisoara, 300041 Timisoara, Romania; gluhovschi.adrian@umft.ro

**Keywords:** abortion, induced, contraception, quality of life, depression, social support

## Abstract

**Background and Objectives**: Abortion on request, contraceptive access barriers, and mental health may jointly shape women’s quality of life (QoL). We examined how abortion history, structural barriers, and psychosocial factors relate to modern contraceptive use, depressive and anxiety symptoms, and QoL among women attending a Romanian tertiary center. **Methods**: We conducted a single-center observational study combining retrospective chart review with an online survey of 200 women aged 18–45 years. Validated instruments (Patient Health Questionnaire-9 [PHQ-9], Generalized Anxiety Disorder-7 [GAD-7], World Health Organization Five-Item Well-Being Index [WHO-5], and World Health Organization Quality of Life–BREF [WHOQOL-BREF]) and indices of access barriers, perceived stigma, and social support were used. Analyses included multivariable regression, structural equation modelling, latent class analysis, and moderation analysis. **Results**: Overall, 55.0% of women reported ≥1 abortion on request. Compared with those without abortion history, they were older (31.2 ± 4.9 vs. 26.8 ± 4.8 years, *p* < 0.001), more often had lower levels of education (51.8% vs. 33.3%, *p* = 0.013), and were less likely to use modern contraception at last intercourse (52.7% vs. 71.1%, *p* = 0.012). PHQ-9 (8.8 ± 4.0 vs. 7.3 ± 4.3) and GAD-7 (7.0 ± 3.2 vs. 5.7 ± 3.4) scores were higher (both *p* = 0.010), while QoL was lower (55.4 ± 8.1 vs. 59.5 ± 7.8, *p* < 0.001). In adjusted models, access barriers (OR per point = 1.3, 95% CI 1.1–1.6), but not abortion history, predicted non-use of modern contraception. QoL correlated strongly with PHQ-9 (r = −0.6) and WHO-5 (r = 0.5; both *p* < 0.001). Latent class analysis identified a “high-barrier, distressed, abortion-experienced” profile with the poorest mental health and QoL. **Conclusions**: Structural access barriers and current depressive and anxiety symptoms, rather than abortion history alone, were key correlates of contraceptive gaps and reduced QoL, underscoring the need for integrated reproductive and mental health care.

## 1. Introduction

Unintended pregnancy and abortion on request remain central public health challenges in many settings, reflecting persistent gaps in contraceptive access, reproductive autonomy, and social support. Recent global modeling suggests that in 2015–2019, there were around 121 million unintended pregnancies per year, with approximately 61% ending in induced abortion—about 73 million abortions annually—disproportionately concentrated in low- and middle-income countries and in settings where abortion is legally restricted [1,2]. Global monitoring by the WHO underscores that restrictive legal regimes do not eliminate abortion; instead, they shift procedures into unsafe conditions that contribute substantially to preventable maternal morbidity and mortality [3,4]. At the same time, unintended pregnancy and abortion have been framed as sensitive indicators of reproductive health and autonomy, shaped by unequal power relations, poverty, and health-system capacity [1,3,5]. Even in contexts where abortion is legally available, structural barriers such as cost, distance, limited service availability, stigma, and fragmented care pathways can delay care and undermine informed contraceptive choice; emerging commentary from psychiatry and public health highlights that tightening legal restrictions is likely to exacerbate psychological distress and widen mental health inequities for historically marginalized groups [4]. Women seeking abortion often present with coexisting stressors including financial insecurity, unstable relationships, or intimate partner violence, which may shape both their reproductive trajectories and mental health.

Access to modern contraception is essential to preventing unintended pregnancy and reducing repeat abortion, yet uptake is influenced by multiple individual and systemic factors. Classical reviews show that when high-quality contraceptive services are expanded, unintended pregnancy generally declines, although trends in abortion can be complex and context-specific [6]. Knowledge of methods, previous experiences of side-effects, fears about infertility, partner attitudes, and religious or cultural norms can all shape contraceptive behavior. At the health-system level, provider bias, stock-outs, limited counseling time, and insufficient integration between family planning and other services may further restrict effective use. Strategic assessments in post-socialist Central and Eastern Europe, including Romania, have described a legacy of reliance on abortion for fertility control, inconsistent adoption of modern methods, and gaps between formal policy and implementation in everyday clinical practice [7]. More recent work from Serbia suggests that, even among women who have experienced induced abortion, intention to use effective contraception is strongly patterned by counseling quality, perceived partner support, and socio-economic status [8]. As a result, women may cycle between periods of non-use, inconsistent use, and reliance on less effective methods.

The experience of abortion on request itself can intersect with mental health in complex ways. For many women, safely provided abortion is primarily associated with relief and resolution of a stressful situation; for others, it may coexist with guilt, ambivalence, or relationship conflict. Longitudinal evidence found that women who received an abortion did not have worse long-term mental health than those denied one; indeed, being denied an abortion was initially associated with higher anxiety, lower self-esteem, and lower life satisfaction, with trajectories converging over five years [9]. Pre-existing depression and anxiety, social isolation, and exposure to violence appear to be stronger determinants of post-abortion distress than the procedure per se [4,9,10]. In Romania, abortion on request is permitted during early pregnancy (up to 14 weeks of gestation), while later procedures are restricted to specific legal/medical grounds. Despite this legal framework, service availability can be uneven across facilities and regions due to organizational barriers and provider refusal, contributing to delayed or foregone caret [10].

Quality of life (QoL) provides a broader lens on the consequences of reproductive experiences, capturing physical, psychological, social, and environmental well-being. The World Health Organization’s quality of life instruments were explicitly designed to operationalize this multidimensional construct across cultures, leading to the development of the WHOQOL and its shorter WHOQOL-BREF form [11,12,13,14]. WHOQOL-BREF has been adapted and validated in multiple European settings, including among medical students in Serbia, supporting its use in Central and Eastern European populations [15]. Standardized mental health and well-being scales complement these QoL measures. The WHO-5 Well-Being Index is a brief, positively worded instrument with good sensitivity to depressive states and change over time [12]. The Patient Health Questionnaire-9 (PHQ-9) and Generalized Anxiety Disorder-7 (GAD-7) are widely used, validated tools for screening and grading the severity of depressive and anxiety symptoms in primary care and community samples [13,14]. When combined, WHOQOL-BREF, WHO-5, PHQ-9, and GAD-7 allow quantification of subjective well-being, functioning, and symptom burden using constructs that are internationally comparable and psychometrically robust [11,12,13,14].

In Romania and other Central and Eastern European settings, evolving health systems and uneven availability of modern contraception may sustain cycles of inconsistent use and reliance on abortion for fertility control. At the same time, mental health symptoms and social support can shape both contraceptive decision-making and perceived quality of life. Few studies in this region have assessed abortion history, contraceptive access barriers, depression/anxiety symptoms, well-being, and multidimensional QoL using standardized instruments within a single protocol [3,5,7].

The present retrospective–cross-sectional study was designed to address this gap by combining anonymized chart review with an online survey incorporating validated mental health and QoL scales. By integrating WHOQOL-BREF, WHO-5, PHQ-9, and GAD-7 within the same protocol [11,12,13,14], we sought to capture both symptom burden and broader well-being in women with diverse reproductive histories. Focusing on women in contact with obstetrics–gynecology, family planning, and psychiatry departments of a tertiary center, we aimed to: (i) compare sociodemographic, reproductive, and mental health characteristics between women with and without a history of abortion on request; (ii) identify factors associated with lack of modern contraception at last intercourse; and (iii) examine the relationships between depressive and anxiety symptoms, subjective well-being, and QoL in this specific Central and Eastern European context.

## 2. Materials and Methods

### 2.1. Study Design and Setting

We conducted a single-center observational study combining a retrospective chart review with a cross-sectional survey. The study took place in a university-affiliated tertiary hospital at the ‘Victor Babeș’ University of Medicine and Pharmacy Timișoara, Romania, which includes obstetrics–gynecology, family planning, and psychiatry departments. These services provide routine antenatal care, abortion on request within legal limits, contraceptive counseling, management of psychiatric disorders, and liaison consultations for women admitted with pregnancy-related complications. This study was approved by the Local Commission of Ethics of the ‘Pius Brînzeu’ Clinical Emergency County Hospital, Timișoara, Romania (approval/protocol no. 107, date: 6 July 2020). The study was conducted in accordance with applicable national regulations and the Declaration of Helsinki. All participants provided informed consent prior to completing the online questionnaire

The retrospective component involved anonymized review of electronic medical records from January 2023 to December 2024. We identified women aged 18–45 years who had at least one documented abortion on request, as well as women attending for contraception counseling, postpartum care, or psychiatric evaluation with no recorded induced abortion. Data were extracted using a standardized template capturing sociodemographic information, reproductive history, and documented contraceptive use at the time of index contact.

The cross-sectional component consisted of an online self-administered questionnaire collected between 1 January 2025 and 30 April 2025. Eligible participants were women aged 18–45 years who had been in contact with any of the three departments during the previous five years and had a valid email address or phone number recorded. Invitations containing a unique survey link were sent by SMS or email. The survey platform enforced one response per participant and stored data on secure institutional servers.

The online questionnaire was anonymous at the response level: no names, national identifiers, or contact details were collected within the survey dataset. Invitation lists (phone/email) were stored separately from responses and were used only for link distribution. Participants who volunteered for optional interviews provided contact details through a separate form not linked to questionnaire responses.

### 2.2. Participants and Recruitment

For the retrospective chart review, all eligible records were screened sequentially. Duplicates and records with missing key variables (age, reproductive status, or outcome of pregnancy) were excluded. The chart review was used primarily to classify women as having vs. not having a history of abortion on request, to confirm the timing and number of abortions, and to document recorded contraceptive status around the time of the procedure or visit.

Sample size rationale. A target sample of 200 completed questionnaires was set based on feasibility and expected precision for key proportions. With *n* = 200, a prevalence near 50% can be estimated with an approximate 95% margin of error of ±7 percentage points. For multivariable models, analyses were prespecified to remain parsimonious (limited predictors per outcome) to reduce overfitting risk.

Given *n* = 200, advanced models (SEM/LCA/moderation) were specified parsimoniously with limited free parameters and were interpreted as exploratory/confirmatory of patterns rather than definitive causal estimates.

For the survey, 320 eligible women were invited, of whom 218 (68.1%) initiated the questionnaire and 200 (62.5% of invited) were selected for inclusion in the final analysis after completing the questionnaires in full. Completeness criteria required valid responses on PHQ-9, GAD-7, WHO-5, WHOQOL-BREF, and key sociodemographic and reproductive items. A subgroup of 26 women volunteering for further contact participated in optional semi-structured interviews (15–20 min) conducted via secure videoconference or phone. Interviews explored perceived barriers to contraception, emotional experience surrounding abortion decisions, interactions with health services, and perceived supports.

Eligibility criteria (survey). Women aged 18–45 years, with prior contact with obstetrics–gynecology, family planning, or psychiatry services within the previous five years, and with a valid email address or phone number. Exclusion criteria included inability to provide informed consent and incomplete responses for primary measures (PHQ-9, GAD-7, WHO-5, WHOQOL-BREF) and key reproductive items.

### 2.3. Measures and Data Collection

The online questionnaire collected sociodemographic variables (age, place of residence, education, employment, living with a partner), reproductive history (parity, number of living children, history and number of abortions on request), and self-reported contraceptive use at last intercourse. For analysis, we defined “modern contraception” as use of hormonal methods, intrauterine devices, implants, or condoms, and “no modern contraception” as withdrawal, periodic abstinence, or no method.

Mental health was assessed using the Patient Health Questionnaire-9 (PHQ-9) for depressive symptoms and the Generalized Anxiety Disorder-7 (GAD-7) scale for anxiety. Both instruments ask participants to rate the frequency of symptoms over the previous two weeks on a 0–3 scale, yielding total scores of 0–27 and 0–21, respectively. We treated PHQ-9 and GAD-7 as continuous variables and also defined “clinically relevant depressive symptoms” as PHQ-9 ≥ 10. Psychological well-being was measured using the WHO-5 index (0–25, higher scores indicating better well-being).

Quality of life was evaluated with the WHOQOL-BREF, which covers physical, psychological, social, and environmental domains. We used the standard scoring algorithm to compute an overall transformed QoL score (0–100, higher scores indicating better QoL). Additional study-specific indices included an “access barrier index” (0–10) derived from Likert items on financial, organizational, and informational obstacles to contraception, a perceived stigma scale (0–10) summarizing feelings of judgment related to abortion or contraceptive use, and a brief social support score (0–10) capturing perceived emotional and practical support from partners, family, and friends.

To reduce information bias, validated instruments with standardized scoring were used. The WHOQOL-BREF Romanian translation is publicly available through WHO resources, and PHQ-9 has been evaluated in Romanian populations. The survey used a one-response-per-participant setting to reduce duplicate entries.

Missing data. The survey platform required completion of PHQ-9, GAD-7, WHO-5, and WHOQOL-BREF prior to submission. Participants with incomplete primary measures were excluded from the analytic sample. Therefore, analyses were conducted as complete-case analyses without imputation.

### 2.4. Statistical Analysis

Analyses were performed in R (R Core Team; R Foundation for Statistical Computing, Vienna, Austria), distributed under the GNU General Public License (GPL). Structural equation modeling used the lavaan package, latent class analysis used poLCA, and figures were produced using ggplot2. Continuous variables were summarized as mean ± standard deviation (SD) and categorical variables as counts and percentages. Group comparisons between women with and without a history of abortion on request were conducted using Welch’s t-tests for continuous variables (to allow for heteroscedasticity) and chi-square tests for categorical variables; Fisher’s exact test was planned for cells with expected counts <5, although this was not required in the final dataset. Two-sided *p*-values <0.05 were considered statistically significant.

The primary outcome for logistic regression was lack of modern contraception at last intercourse (binary variable: 1 = no modern method; 0 = modern method). Predictors included history of abortion on request and the access barrier index. Adjusted odds ratios (ORs) with 95% confidence intervals (CI) were derived from model coefficients. Model fit was assessed using the likelihood ratio test and pseudo-R^2^ statistics.

Pearson correlation coefficients quantified associations between WHOQOL-BREF overall QoL and PHQ-9, GAD-7, WHO-5, access barrier index, stigma, and social support scores. A multivariable linear regression model was constructed with overall QoL as the dependent variable and PHQ-9, GAD-7, abortion history, access barrier index, and social support as predictors. Regression coefficients (β) with 95% CI and *p*-values were reported, and model performance was summarized using R^2^. Assumptions of linearity, homoscedasticity, and normality of residuals were evaluated visually and deemed acceptable.

SEM was used to evaluate a prespecified conceptual model in which access barriers and abortion history relate to depressive symptoms (PHQ-9), anxiety symptoms (GAD-7), and overall QoL, allowing estimation of direct and indirect (mediated) effects within a single framework. Models were estimated in R using lavaan with maximum likelihood estimation, and indirect effects were assessed using bootstrap confidence intervals. Model fit was evaluated using the comparative fit index (CFI), root mean square error of approximation (RMSEA), and standardized root mean square residual (SRMR). We interpreted CFI ≥ 0.90–0.95, RMSEA ≤ 0.06–0.08, and SRMR ≤ 0.08 as indicating acceptable fit.

## 3. Results

Women with a history of abortion on request were older and more frequently had low education and higher parity compared with women without abortion history. Modern contraception at last intercourse was less common among women with abortion history. Full sociodemographic and reproductive comparisons are provided in Table 1.

PHQ-9 and GAD-7 scores were higher and WHOQOL-BREF overall QoL scores were lower among women with abortion history, while WHO-5 did not differ significantly between groups (Table 2).

In adjusted logistic regression, the access barrier index was associated with a lack of modern contraception at last intercourse, whereas abortion history was not statistically significant (Table 3).

Table 4 focuses on the subset of 110 women with a history of abortion on request and explores whether mental health, well-being, and perceived stigma differ according to the number of abortion episodes. Two groups were defined: women with a single abortion (*n* = 64) and those with multiple abortions (≥2, *n* = 46). Contrary to expectations that repeated abortions might be associated with greater psychological burden, depressive and anxiety symptom scores were broadly similar between groups. Mean PHQ-9 scores were 9.2 ± 3.9 in the single-abortion group and 8.2 ± 4.2 in the multiple-abortion group (*p* = 0.183), and GAD-7 scores were 7.1 ± 3.1 vs. 6.8 ± 3.2 (*p* = 0.674), indicating no statistically significant differences. WHO-5 well-being scores were slightly higher among women with multiple abortions (13.3 ± 3.6 vs. 12.7 ± 3.2, *p* = 0.355), but the difference was small and not significant. Overall QoL scores on WHOQOL-BREF were also closely aligned (55.0 ± 8.2 vs. 55.9 ± 8.1, *p* = 0.593), suggesting that the perceived impact on everyday functioning, relationships, and environment did not worsen with increasing number of abortions in this sample. Finally, perceived stigma scores were essentially identical (4.1 ± 1.5 vs. 4.1 ± 1.6, *p* = 0.980).

Table 5 displays bivariate correlations between overall QoL and several psychosocial variables. Depressive symptoms showed the strongest association: PHQ-9 scores correlated moderately and negatively with QoL (r = −0.6, *p* < 0.001), indicating that higher levels of depressed mood, anhedonia, and related symptoms are robustly linked to lower perceived quality of life across physical, psychological, social, and environmental domains. Anxiety symptoms, as measured by GAD-7, also exhibited a negative correlation with QoL (r = −0.4, *p* < 0.001). In contrast, WHO-5 psychological well-being scores correlated positively with QoL (r = 0.5, *p* < 0.001). The access barrier index, perceived stigma, and social support displayed only weak and statistically non-significant correlations with QoL (r values around ±0.1, *p* > 0.19).

Table 6 shows a multivariable linear regression model for overall QoL (WHOQOL-BREF, 0–100). Both depressive and anxiety symptoms are strong independent predictors of lower QoL: each one-point increase in PHQ-9 is associated with a 1.1-point decrease in QoL (β = −1.1, 95% CI −1.3 to −0.9, *p* < 0.001), and each one-point increase in GAD-7 with a 1.0-point decrease (β = −1.0, 95% CI −1.2 to −0.7, *p* < 0.001). History of abortion on request has a modest, non-significant negative association with QoL (β = −1.2, 95% CI −3.0 to 0.6, *p* = 0.183), while access barriers and social support scores show small, non-significant coefficients near zero (β around −0.2, all *p* > 0.37). The model explains half of the variance in QoL (R^2^ = 0.5; overall *p* < 0.001).

Table 7 summarizes standardized direct paths from a structural equation model (SEM) linking access barriers and abortion history to mental health and QoL. Higher access barriers are associated with greater depressive (PHQ-9; β = 0.4, *p* = 0.001) and anxiety (GAD-7; β = 0.3, *p* = 0.004) symptoms, and with lower QoL via a significant direct path (β = −0.2, *p* = 0.016). Similarly, abortion history shows significant positive paths to both PHQ-9 (β = 0.2, *p* = 0.027) and GAD-7 (β = 0.2, *p* = 0.032), suggesting higher symptom burden in women with abortion experience. Mental health symptoms in turn have strong negative paths to QoL: PHQ-9 (β = −0.5, *p* < 0.001) and GAD-7 (β = −0.2, *p* = 0.007) each independently reduce overall QoL. The direct path from abortion history to QoL is small and non-significant (β = −0.1, *p* = 0.184).

Table 8 reports the indirect effects of access barriers and abortion history on overall QoL through depressive and anxiety symptoms. Access barriers show a substantial negative indirect effect on QoL (standardized β = −0.3, 95% CI −0.4 to −0.2, *p* < 0.001), indicating that a sizeable portion of their impact operates via increasing PHQ-9 and GAD-7 scores rather than solely through direct pathways. Abortion history also exhibits a negative indirect effect on QoL (β = −0.2, 95% CI −0.3 to −0.1, *p* = 0.009), again mediated through elevated depressive and anxiety symptoms.

Table 9 presents explained variance (R^2^) for the endogenous variables and overall model fit indices for the SEM. The model accounts for 30% of the variance in depressive symptoms (PHQ-9 R^2^ = 0.3) and 20% in anxiety symptoms (GAD-7 R^2^ = 0.2), indicating moderate explanatory power for mental health outcomes. For overall QoL, the model performs particularly well, explaining 60% of the variance (WHOQOL-BREF R^2^ = 0.6), consistent with the strong paths from depression, anxiety, and access barriers observed in earlier tables. Global fit indices suggest an acceptable to good fit: CFI = 0.94 (close to the conventional 0.95 threshold), RMSEA = 0.07, and SRMR = 0.06, all within ranges typically interpreted as indicating reasonably well-fitting structural models.

Table 10 summarizes a three-class latent class analysis (LCA) of reproductive–mental health profiles. Class 1 (“well-supported contraceptive users,” 36.2% of the sample) shows low probability of abortion history (31.8%), high likelihood of modern contraception at last intercourse (86.4%), relatively low PHQ-9 (6.1) and GAD-7 (4.7) scores, high WHO-5 well-being (15.2), the highest QoL (WHOQOL-BREF 61.7), low access barriers (2.6), and strong social support (8.3). Class 2 (“high-barrier, distressed, abortion-experienced,” 29.7%) is characterized by very high probability of abortion history (82.9%), low modern contraception use (39.7%), high depressive (PHQ-9 11.6) and anxiety (GAD-7 9.3) scores, low well-being (WHO-5 10.8), the lowest QoL (50.9), very high access barriers (7.8), and weak social support (3.7). Class 3 (“young transitional mixed profile,” 34.1%) occupies an intermediate position, with abortion history probability 57.4%, modern contraception 55.2%, moderate symptom scores (PHQ-9 7.9; GAD-7 6.4), WHO-5 13.5, QoL 57.4, barriers 4.9, and support 6.1. The 3-class solution is supported by the lowest BIC (2123.4) and high entropy (0.8), indicating good separation between classes.

Table 11 reports a moderation analysis evaluating whether social support buffers the association between abortion history and overall QoL. Depressive symptoms (PHQ-9) remain a strong negative predictor of QoL (β = −0.9, 95% CI −1.1 to −0.7, *p* < 0.001), while social support shows a positive main effect (β = 1.3, 95% CI 0.8 to 1.9, *p* < 0.001), indicating that each additional point on the 0–10 support scale is associated with roughly a 1.3-point increase in QoL. Abortion history is associated with substantially lower QoL (β = −7.3, 95% CI −11.1 to −3.4, *p* = 0.001), but the significant interaction term (abortion history × social support: β = 0.9, 95% CI 0.3 to 1.6, *p* = 0.006) suggests that higher social support attenuates this negative effect. The model explains 60% of the variance in QoL (R^2^ = 0.6), and inclusion of the interaction improves explained variance by ΔR^2^ = 0.04 (likelihood ratio test *p* = 0.013).

Table 12 details the conditional effect of abortion history on overall QoL at different levels of social support, derived from the moderation model. At low social support (2 points below the sample mean), having an abortion history is associated with a pronounced reduction in QoL (β = −9.1, 95% CI −13.4 to −4.8), indicating a roughly 9-point lower WHOQOL-BREF score compared with women without abortion history at the same low support level. At average support, the negative effect remains substantial (β = −7.3, 95% CI −11.1 to −3.4), while at high support (2 points above the mean) the effect size attenuates to β = −5.6 (95% CI −10.1 to −1.2), as seen in Table 11.

**Figure 1:**
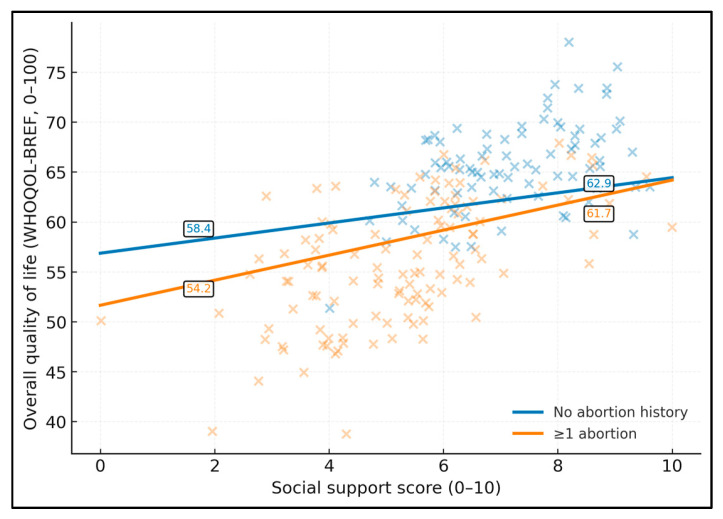
Predicted overall quality of life (WHOQOL-BREF, 0–100) by social support score (0–10), stratified by abortion history (≥1 vs. none), from a linear regression model adjusted for PHQ-9 and including an abortion history × social support interaction.

Higher social support was associated with higher QoL, with a modest buffering of the abortion–QoL difference at higher support levels (Figure 1). Among women without a history of abortion, predicted QoL increases from approximately 58.9 at a low social support score of 2 to 69.7 at a support score of 8. In contrast, for women with at least one abortion on request, predicted QoL rises from 52.4 at support 2 to 63.3 at support 8, indicating a consistently lower QoL at comparable support levels but a similar positive slope.

**Figure 2:**
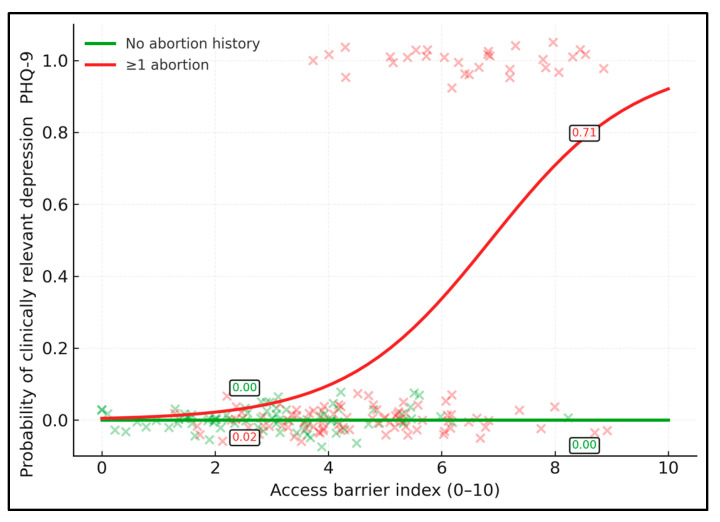
Predicted probability of clinically relevant depressive symptoms (PHQ-9 ≥ 10) by access barrier index (0–10), stratified by abortion history (≥1 vs. none), from a logistic regression model including an interaction term.

Higher access barriers were associated with higher predicted probability of PHQ-9 ≥ 10, particularly among women with abortion history (Figure 2). Among women without abortion history, the predicted probability of depression increases from 0.13 at a barrier index of 2 to 0.41 at a barrier index of 8. For women with abortion history, the corresponding probabilities rise more steeply, from 0.23 at barrier 2 to 0.66 at barrier 8, indicating a marked amplification of risk in the context of structural obstacles to contraception. At very low barriers, depression risk remains non-negligible but moderate in both groups, whereas at high barriers the probability of clinically relevant depression approaches two-thirds among women who have undergone abortion on request.

**Figure 3:**
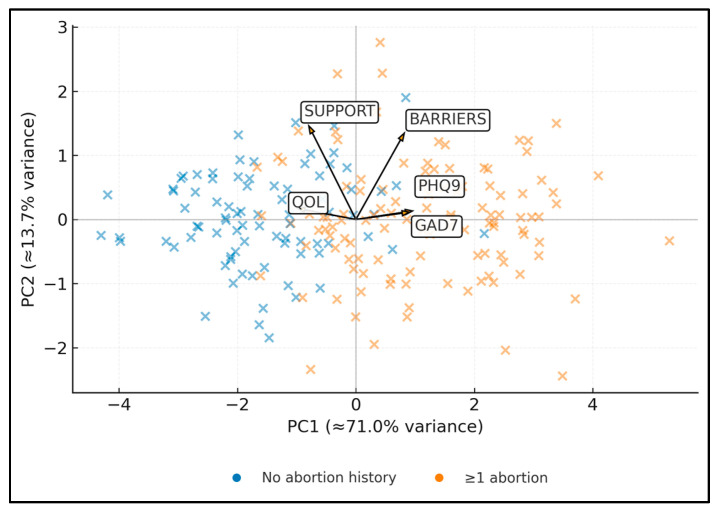
Principal component analysis (PCA) biplot of PHQ-9, GAD-7, overall QoL, access barriers, and social support, with participants labeled by abortion history (≥1 vs. none). PC1 and PC2 explain 71.0% and 13.7% of variance, respectively.

PC1 primarily reflected a distress/barrier gradient opposed to QoL/support (Figure 3). The first principal component (PC1) explained approximately 71.0% of total variance, whereas PC2 accounted for 13.7%, indicating that PC1 captures the dominant gradient of risk and resilience. Women with a history of abortion on request tended to cluster on the positive side of PC1 (mean PC1 score 1.1) compared with those without abortion history (mean PC1 score −1.6), suggesting a systematic shift toward higher symptom burden and lower QoL in this group. These findings indicate that worse mental health and greater barriers align with lower QoL and weaker support. Along PC2, there is a modest differentiation between anxiety and depression versus barriers and support, hinting at secondary patterns not captured by simple bivariate contrasts.

**Figure 4:**
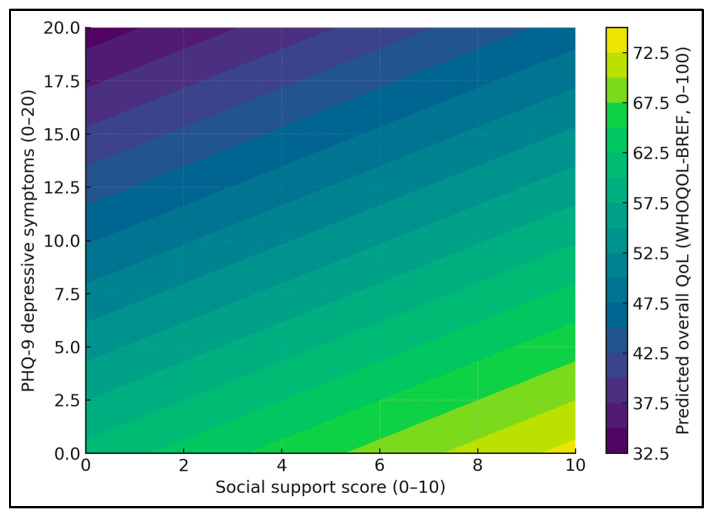
Predicted overall quality of life (WHOQOL-BREF, 0–100) as a function of depressive symptoms (PHQ-9) and social support among women with ≥1 abortion on request, based on the multivariable model including the interaction term.

QoL decreased with increasing PHQ-9 and increased with higher support within the abortion-history subgroup (Figure 4). In women with mild depressive symptoms (PHQ-9≈5), predicted QoL increases from about 56.5 at low support (score 2) to 64.1 at high support (score 8), a gain of roughly 7.6 points. In contrast, among women with more severe depressive symptoms (PHQ-9≈15), QoL is markedly lower overall, rising from only 42.9 at support 2 to 50.4 at support 8; the absolute improvement is similar (~7.5 points), but even with strong support the predicted QoL remains substantially below that of mildly depressed women.

## 4. Discussion

### 4.1. Analysis of Findings

This study addressed three objectives: (i) to compare women with vs. without abortion history; (ii) to identify correlates of non-use of modern contraception; and (iii) to quantify relationships between depression/anxiety, well-being, and QoL. The main findings were that access barriers were associated with contraceptive non-use, and depressive/anxiety symptoms were the strongest independent correlates of lower QoL, while abortion history showed limited direct associations after adjustment.

Women in our sample who had undergone abortion on request were older, more often multiparous, and more likely to report low education and non-use of modern contraception at last intercourse, a pattern that resonates with prior work from post-socialist Europe showing that abortion is frequently embedded in trajectories of socio-economic vulnerability and incomplete contraceptive transitions [7,8]. In Johnson et al.’s strategic assessment from Romania, women relying on abortion for fertility control often reported fragmented counseling and inconsistent access to modern methods, particularly in lower-education and rural groups [7]. Similar dynamics are visible in postabortion cohorts from Ethiopia and Pakistan, where educational level, partner communication, and perceived quality of counseling were key determinants of postabortion contraceptive uptake, while cost, stock-outs, and fear of side effects discouraged effective method use [16,17]. Our finding that an incremental increase in access barriers substantially raised the odds of lacking modern contraception, whereas abortion history itself was not an independent predictor, is therefore consistent with the broader literature emphasizing that structural and informational obstacles—rather than women’s prior abortion experience per se—are central drivers of non-use and method discontinuation in diverse settings [6,7,8,16,17].

The mental health and QoL differences observed between women with and without a history of abortion on request require careful interpretation in light of existing longitudinal evidence. We found modestly higher depressive and anxiety symptom scores and lower overall QoL among women with abortion history, but multivariable models and SEM indicated that these associations were largely mediated by current symptom burden and access barriers rather than abortion history acting as an independent determinant. This pattern aligns with large cohort re-analyses from the United States and Europe showing that once pre-existing mental health problems, violence exposure, and socio-economic adversity are accounted for, abortion history is not a strong independent predictor of subsequent mental disorders [18,19]. Rocca et al.’s Turnaway analyses similarly reported that women who obtained abortions generally experienced declining negative emotions and high “decision rightness” over three years, with no evidence of worsening mental health relative to women denied care [9,20]. Our cross-sectional data therefore appear more consistent with a “common risk factors” framework—where the same social, relational, and psychological vulnerabilities increase both the likelihood of needing an abortion and of experiencing depression or anxiety—than with models positing abortion itself as a major causal driver of psychopathology [18,19].

Within the subgroup of women who had at least one abortion, the absence of clear gradients in depressive symptoms, anxiety, QoL or perceived stigma by number of abortion episodes also dovetails with this common-risk-factors perspective. Steinberg and colleagues, re-analyzing National Comorbidity Survey data, found that apparent dose–response relationships between multiple abortions and poor mental health largely disappeared after controlling for prior mental health and adversity [18]. Our latent class analysis adds nuance by showing that the most distressed profile (“high-barrier, distressed, abortion-experienced”) was characterized not just by abortion history but by concentrated structural barriers, low social support, and high psychosocial symptom load, whereas a sizeable subgroup of abortion-experienced women clustered with “well-supported contraceptive users” and reported relatively favorable mental health and QoL. This pattern echoes recent work with the Psychosocial Burden among people Seeking Abortion Scale (PB-SAS), which identified structural challenges, decision difficulty, lack of autonomy, and concerns about others’ reactions as the key domains associated with elevated stress, anxiety, and depressive symptoms at the time of seeking abortion [21]. Our relatively modest direct associations between perceived stigma and QoL, but stronger indirect effects via depressive and anxiety symptoms in the SEM, are broadly compatible with the abortion-stigma literature, where internalized stigma and anticipated judgment amplify distress primarily through their impact on self-esteem, social isolation, and help-seeking rather than through a simple one-to-one link with QoL scores [10,22].

The SEM and moderation analyses further highlight how structural and psychosocial determinants intersect. Access barriers showed a significant direct negative path to QoL and substantial indirect effects mediated by higher PHQ-9 and GAD-7 scores, even though their simple bivariate correlation with QoL was weak. This suggests that barriers may function as “upstream” stressors whose effects emerge most clearly when the broader network of symptoms and resources is modeled jointly, consistent with Biggs et al.’s finding that structural challenges (travel, scheduling, difficulty finding a provider) formed the dominant component of psychosocial burden among people seeking abortion and were strongly linked to worse mental health at the time of care seeking [21]. Our latent classes map onto this logic: the class with the highest barrier scores also exhibited the highest PHQ-9/GAD-7 scores and the lowest QoL, whereas women with lower barriers and stronger social support reported substantially better outcomes despite similar reproductive histories. Together with longitudinal evidence showing that rigorously measured structural restrictions—such as mandated waiting periods and gestational limits—can increase stress and delay care without improving mental health outcomes [4,19,21], these findings argue for prioritizing barrier reduction and integrated mental health support over policies that focus narrowly on abortion exposure.

Finally, the moderating role of social support in our data is in line with both abortion-specific and broader perinatal mental health literature. We found that higher social support was independently associated with better QoL and attenuated (though did not eliminate) the QoL gap between women with and without abortion history, with the largest absolute benefits among those reporting low to moderate support. This mirrors results from the Turnaway Study analysis of perceived stress and emotional social support, in which women denied abortions initially reported higher stress but similar levels of emotional social support compared with those who obtained abortions, and trajectories of stress converged over time as support networks adapted [23]. More generally, studies in pregnant and postpartum populations consistently show that stronger social support is linked to better health-related QoL and lower depressive symptoms, while low support predicts poorer mental and physical well-being [24]. Our findings extend this evidence to women with diverse reproductive histories in a Central and Eastern European context, underscoring that efforts to strengthen partner, family, and community support—and to embed systematic mental health and social support assessments into abortion and contraceptive services—may be crucial in mitigating the psychosocial impact of unintended pregnancy, abortion, and ongoing barriers to reproductive care.

The findings support practical steps to reduce contraceptive gaps and improve mental health–related quality of life in Romanian tertiary-care centers [25,26,27]. First, routine visits in obstetrics–gynecology and family planning could incorporate brief, standardized screening for depressive and anxiety symptoms (PHQ-9/GAD-7) paired with a clear referral pathway to mental health services. Second, barrier-reduction strategies—transparent counseling on method options, streamlined appointments, predictable costs, and consistent availability of modern methods—may yield larger gains than focusing on abortion history alone. Third, the identified high-barrier/distressed subgroup suggests value in targeted, multidisciplinary interventions (contraceptive counseling + psychosocial support + social assistance navigation) rather than uniform, one-size-fits-all care. Future multicenter studies should test whether structured counseling programs and integrated care pathways improve modern contraceptive uptake and QoL while reducing symptom burden.

### 4.2. Study Limitations

This study has several limitations. Its cross-sectional design precludes causal inference regarding the temporal relationships between abortion history, access barriers, mental health, and QoL. The single tertiary-center setting and requirement for valid contact details may limit generalizability, favoring women more engaged with formal health services and potentially under-representing those most marginalized. Mental health, QoL, stigma, barriers, and support were assessed using self-report instruments and are vulnerable to recall and social desirability bias. This is particularly relevant for sensitive variables such as abortion history and perceived access barriers, which may be stigmatized and therefore susceptible to underreporting or non-disclosure, potentially attenuating observed associations. Although we used validated scales and multivariable models, residual confounding by unmeasured factors—such as intimate partner violence, childhood adversity, or detailed socio-economic indicators—cannot be excluded. The moderate sample size (*n* = 200) imposes constraints on the complexity of structural equation and latent class models, raising the possibility of overfitting and model instability. Selection mechanisms may have biased estimates toward women engaged with formal care and reachable by phone/email, potentially underestimating barriers and distress in more marginalized groups. Social desirability and recall bias could lead to underreporting of abortion history and barriers, which would generally bias group differences and associations toward the null. Given the multiplicity of analytic approaches (regression, SEM, LCA, moderation), results should be interpreted as complementary and hypothesis-generating rather than definitive causal evidence. Finally, we did not capture gestational age, time since abortion, or characteristics of abortion care (e.g., counselling quality, provider attitude), which may further shape mental health trajectories and reproductive decision-making.

## 5. Conclusions

In this Central and Eastern European tertiary care setting, more than half of participating women reported at least one abortion on request, and this group showed lower modern contraceptive use, slightly higher depressive and anxiety symptoms, and poorer QoL than women without abortion history. However, once structural and psychosocial factors were taken into account, access barriers and current symptom burden emerged as the principal correlates of both contraceptive gaps and impaired QoL, whereas abortion history itself played a more modest and largely indirect role. Advanced modelling highlighted a distinct subgroup of women characterized by combined high barriers, prior abortion, low social support, and marked psychological distress. These results support a shift from pathologizing abortion per se toward addressing the broader structural and interpersonal contexts in which unintended pregnancy and abortion occur. Integrating high-quality contraceptive counselling with systematic mental health assessment and targeted support for women facing multiple barriers may improve both reproductive autonomy and mental health-related quality of life.

## Figures and Tables

**Table 1:** Sociodemographic and reproductive characteristics by history of abortion on request.

Variable	≥1 Abortion on Request (*n* = 110)	No Abortion on Request (*n* = 90)	*p*-Value
Age, years	31.2 ± 4.9	26.8 ± 4.8	<0.001
Rural residence, %	29.1	26.7	0.825
Low education, %	51.8	33.3	0.013
Living with partner, %	71.8	55.6	0.025
Parity, *n*	1.5 ± 1.2	0.8 ± 1.0	<0.001
Living children, *n*	1.3 ± 1.3	0.7 ± 1.0	<0.001
Modern contraception at last intercourse, %	52.7	71.1	0.012

Values are mean ± SD or %. Abbreviations: *n*, number; SD, standard deviation; %, percentage.

**Table 2:** Mental health and quality of life scores by history of abortion on request.

Outcome	≥1 Abortion on Request (*n* = 110)	No Abortion on Request (*n* = 90)	*p*-Value
PHQ-9 score	8.8 ± 4.0	7.3 ± 4.3	0.01
GAD-7 score	7.0 ± 3.2	5.7 ± 3.4	0.01
WHO-5 well-being score	12.9 ± 3.4	13.7 ± 3.2	0.108
WHOQOL-BREF overall QoL (0–100)	55.4 ± 8.1	59.5 ± 7.8	<0.001

Values are mean ± SD. Abbreviations: PHQ-9, Patient Health Questionnaire-9; GAD-7, Generalized Anxiety Disorder-7; WHO-5, World Health Organization Five-Item Well-Being Index; WHOQOL-BREF, World Health Organization Quality of Life—BREF; QoL, quality of life; SD, standard deviation.

**Table 3:** Logistic regression for lack of modern contraception at last intercourse.

Predictor	Adjusted OR	95% CI	*p*-Value
History of abortion on request (yes vs. no)	1.7	0.9–3.1	0.109
Access barrier index (per 1-point increase)	1.3	1.1–1.6	0.001

Outcome: no modern contraception at last intercourse (1 = yes, 0 = no). Model pseudo-R^2^ = 7.0%; likelihood ratio test *p* < 0.001. Abbreviations: OR, odds ratio; CI, confidence interval; R^2^, coefficient of determination.

**Table 4:** Mental health, well-being, quality of life, and perceived stigma among women with a history of abortion on request, by number of abortions (one vs. ≥2).

Outcome	One Abortion (*n* = 64)	≥2 Abortions (*n* = 46)	*p*-Value
PHQ-9 score	9.2 ± 3.9	8.2 ± 4.2	0.183
GAD-7 score	7.1 ± 3.1	6.8 ± 3.2	0.674
WHO-5 well-being score	12.7 ± 3.2	13.3 ± 3.6	0.355
WHOQOL-BREF overall QoL (0–100)	55.0 ± 8.2	55.9 ± 8.1	0.593
Perceived stigma score (0–10)	4.1 ± 1.5	4.1 ± 1.6	0.98

Values are mean ± SD. Abbreviations: PHQ-9, Patient Health Questionnaire-9; GAD-7, Generalized Anxiety Disorder-7; WHO-5, World Health Organization Five-Item Well-Being Index; WHOQOL-BREF, World Health Organization Quality of Life—BREF; QoL, quality of life; SD, standard deviation.

**Table 5:** Pearson correlations between overall QoL and key psychosocial variables.

Predictor	Pearson r	*p*-Value
PHQ-9 score	−0.6	<0.001
GAD-7 score	−0.4	<0.001
WHO-5 well-being score	0.5	<0.001
Access barrier index	−0.1	0.346
Perceived stigma score	−0.1	0.295
Social support score	0.1	0.196

Outcome variable: WHOQOL-BREF overall QoL (0–100). Abbreviations: PHQ-9, Patient Health Questionnaire-9; GAD-7, Generalized Anxiety Disorder-7; WHO-5, World Health Organization Five-Item Well-Being Index; WHOQOL-BREF, World Health Organization Quality of Life—BREF; QoL, quality of life; r, Pearson correlation coefficient.

**Table 6:** Linear regression for overall quality of life (WHOQOL-BREF).

Predictor	β Coefficient	95% CI	*p*-Value
PHQ-9 score (per 1-point increase)	−1.1	−1.3–−0.9	<0.001
GAD-7 score (per 1-point increase)	−1.0	−1.2–−0.7	<0.001
History of abortion on request (yes vs. no)	−1.2	−3.0–0.6	0.183
Access barrier index (per 1-point increase)	−0.2	−0.6–0.3	0.459
Social support score (per 1-point increase)	−0.2	−0.7–0.3	0.374

Outcome: WHOQOL-BREF overall QoL (0–100). Abbreviations: PHQ-9, Patient Health Questionnaire-9; GAD-7, Generalized Anxiety Disorder-7; QoL, quality of life; β, regression coefficient; CI, confidence interval; R^2^, coefficient of determination.

**Table 7:** Structural equation model (SEM): Access barriers, abortion history, mental health, and overall: Panel A. Direct paths (standardized coefficients).

Path	Standardized β	*p*-Value
Access barriers → PHQ-9	0.4	0.001
Access barriers → GAD-7	0.3	0.004
Abortion history (yes vs. no) → PHQ-9	0.2	0.027
Abortion history (yes vs. no) → GAD-7	0.2	0.032
PHQ-9 → WHOQOL-BREF overall QoL	−0.5	<0.001
GAD-7 → WHOQOL-BREF overall QoL	−0.2	0.007
Access barriers → WHOQOL-BREF overall QoL (direct effect)	−0.2	0.016
Abortion history → WHOQOL-BREF overall QoL (direct effect)	−0.1	0.184

Abbreviations: SEM, structural equation model; PHQ-9, Patient Health Questionnaire-9; GAD-7, Generalized Anxiety Disorder-7; WHOQOL-BREF, World Health Organization Quality of Life—BREF; QoL, quality of life; β, standardized path coefficient.

**Table 8:** Indirect effects on overall QoL (via PHQ-9 and GAD-7).

Indirect Path	Standardized β	95% CI	*p*-Value
Access barriers → QoL (via PHQ-9 and GAD-7)	−0.3	−0.4 to −0.2	<0.001
Abortion history → QoL (via PHQ-9 and GAD-7)	−0.2	−0.3 to −0.1	0.009

Abbreviations: QoL, quality of life; PHQ-9, Patient Health Questionnaire-9; GAD-7, Generalized Anxiety Disorder-7; β, standardized indirect effect; CI, confidence interval.

**Table 9:** Explained variance and model fit.

Endogenous Variable	R^2^
PHQ-9	0.3
GAD-7	0.2
WHOQOL-BREF overall QoL	0.6

Model fit indices: CFI = 0.94, RMSEA = 0.07, SRMR = 0.06; Abbreviations: PHQ-9, Patient Health Questionnaire-9; GAD-7, Generalized Anxiety Disorder-7; WHOQOL-BREF, World Health Organization Quality of Life—BREF; QoL, quality of life; R^2^, coefficient of determination; CFI, Comparative Fit Index; RMSEA, Root Mean Square Error of Approximation; SRMR, Standardized Root Mean Square Residual.

**Table 10:** Latent class analysis (3-class solution) of reproductive–mental health profiles using abortion history, modern contraception use, mental health scores, QoL, access barriers, and social support.

Variable	Class 1—“Well-Supported Contraceptive Users”	Class 2—“High-Barrier, Distressed, Abortion-Experienced”	Class 3—“Young Transitional Mixed Profile”
Approximate class size, %	36.2	29.7	34.1
Probability of abortion history, %	31.8	82.9	57.4
Probability of modern contraception at last intercourse, %	86.4	39.7	55.2
PHQ-9 mean score	6.1	11.6	7.9
GAD-7 mean score	4.7	9.3	6.4
WHO-5 well-being score	15.2	10.8	13.5
WHOQOL-BREF overall QoL (0–100)	61.7	50.9	57.4
Access barrier index (0–10)	2.6	7.8	4.9
Social support score (0–10)	8.3	3.7	6.1

Model fit: BIC = 2123.4; entropy = 0.80. Abbreviations: LCA, latent class analysis; PHQ-9, Patient Health Questionnaire-9; GAD-7, Generalized Anxiety Disorder-7; WHO-5, World Health Organization Five-Item Well-Being Index; WHOQOL-BREF, World Health Organization Quality of Life—BREF; QoL, quality of life; BIC, Bayesian Information Criterion.

**Table 11:** Moderation analysis: social support buffering the association between abortion history and overall QoL.

Predictor	β Coefficient	95% CI	*p*-Value
PHQ-9 score (per 1-point increase)	−0.9	−1.1 to −0.7	<0.001
Social support score (per 1-point increase)	1.3	0.8 to 1.9	<0.001
Abortion history (1 = ≥1 abortion, 0 = none)	−7.3	−11.1 to −3.4	0.001
Abortion history × Social support	0.9	0.3 to 1.6	0.006

Linear regression model with interaction term; outcome = WHOQOL-BREF overall QoL (0–100); Model R^2^ = 0.6; inclusion of the interaction term improved explained variance by ΔR^2^ = 0.04 (likelihood ratio test *p* = 0.013). Outcome: WHOQOL-BREF overall QoL (0–100). Abbreviations: PHQ-9, Patient Health Questionnaire-9; QoL, quality of life; β, regression coefficient; CI, confidence interval; R^2^, coefficient of determination.

**Table 12:** Conditional effect of abortion history on QoL at different levels of social support.

Social Support Level *	Effect of Abortion History on QoL (β)	95% CI
Low (2 points below mean)	−9.1	−13.4 to −4.8
Mean	−7.3	−11.1 to −3.4
High (2 points above mean)	−5.6	−10.1 to −1.2

* Support level defined relative to the sample mean on the 0–10 scale. Abbreviations: QoL, quality of life; β, regression coefficient; CI, confidence interval.

## Data Availability

The data presented in this study are available on request from the corresponding author due to privacy and ethical reasons.
